# Supplementary material for: Isolation, growth, and nitrogen fixation rates of the Hemiaulus-Richelia (diatom-cyanobacterium) symbiosis in culture
Source: PeerJ. 2020 Oct 8;8:e10115. doi: 10.7717/peerj.10115 (PMC7548074; doi:10.7717/peerj.10115)
Supplement: Supplemental Information 3 [file peerj-08-10115-s003.pdf]

Supplemental information

The Jassby-Platt hyperbolic tangent function (Jassby and Platt 1976) is commonly used to fit data that exhibits saturation kinetics to light gradient but does not exhibit photoinhibition. For gross photosynthesis, it has the form of:

Equation 1: $P_{grossB} = P_{Bm} \tanh(\alpha I / P_{Bm})$

Where:
P_B = biomass-normalized gross productivity
P_{Bm} = maximum biomass-normalized gross productivity
α = initial light-limited slope of the curve
I = irradiance

P_{Bm} is a curve-fit generated term. In modern usage, I (often an energy-based measurement in the older literature) has been replaced with E, a quanta based measurement (Sakshaug et al. 1997). When net photosynthesis is modelled, a respiration term is included to describe a non-zero intercept:

Equation 2: $P_{netB} = P_{grossB} - R_B$

Where:
P_{netB} = biomass-normalized net productivity
R_B = biomass-normalized respiration

Leading to:

Equation 3: $P_{netB} = [P_{Bm} \tanh(\alpha E / P_{Bm})] - R_B$

These equations are used for light-dependent nitrogen-fixation and growth rates of the *H. hauckii* DDA. For N_2 fixation, the diel response and time dependent dark nitrogen fixation made inclusion of a dark respiration term meaningless in the context of the experiments and Equation 1 was considered sufficient for the curve fit. For growth rates, compensation light intensity (E_c) is a useful term and Equation 2 was used.

N_2 fixation: Equation 4: $N_F = N_{Fmax} \tanh(\alpha E / N_{Fmax})$

Where:
N_F = heterocyst-normalized N_2 fixation
N_{Fmax} = heterocyst-normalized maximum N_2 fixation
α = initial light-limited slope of the curve
E = photosynthetically available radiation as photon flux

Growth rate: Equation 5: $\mu = [\mu_{max} \tanh(\alpha I / \mu_{max})] - \mu_d$

Where:
μ = growth rate
μ_{max} = maximum growth rate
α = initial light-limited slope of the curve
E = photosynthetically available radiation as photon flux
μ_d = curve-fit growth rate at E=0. Graphically, the y-intercept of α

E_c is calculated from $\mu_{d/}$ α (x-intercept of α). E_k is calculated from μ_{max}/ α (Sakshaug et al. 1997). Parameter for these curves are in Table S1.

Reference

Sakshaug E, Bricaud A, Dandonneau Y, Falkowski PG, Kiefer DA, Legendre L, Morel A, Parslow J, and Takahashi M. 1997. Parameters of photosynthesis: definitions, theory and interpretation of results. *Journal of Plankton Research* 19:1637-1670. 10.1093/plankt/19.11.1637
